# Intestinal Malrotation With Pericecal Hernia Causing a Small Bowel Obstruction: A Case Report

**DOI:** 10.7759/cureus.115054

**Published:** 2026-08-23

**Authors:** Ankith Reddy Narra, Nagarjun Nelluri, Gandhala Shlaghya, Diya Venkata Sai Dheeraj, Sreedhar Rao Kota, Kishore Abuji

**Affiliations:** 1 General Surgery, Employees' State Insurance Corporation (ESIC) Medical College, Hyderbad, IND; 2 General Surgery, Employees' State Insurance Corporation (ESIC) Medical College, Hyderabad, IND; 3 Vascular Surgery, Sree Chitra Thirunal Institute for Medical Sciences and Technology, Thiruvananthapuram, IND

**Keywords:** congenital bowel malrotation, intestinal malrotation, pericecal hernia, small bowel obstruction, strangulated internal hernia

## Abstract

Congenital intestinal malrotation is an uncommon developmental anomaly that rarely presents in adulthood. Its coexistence with a pericecal internal hernia and acute midgut volvulus is exceptionally rare, making preoperative diagnosis challenging. We report the case of a 57-year-old man who presented with severe abdominal pain, progressive abdominal distension, vomiting, and obstipation, accompanied by hypovolemic shock. Laboratory investigations revealed leukocytosis, metabolic acidosis, and elevated serum lactate levels. Contrast-enhanced computed tomography demonstrated distal small bowel obstruction with bowel wall thickening and free intraperitoneal fluid, suggestive of strangulation. Emergency exploratory laparotomy revealed congenital intestinal malrotation with the duodenojejunal junction on the right side, an incarcerated pericecal internal hernia, and acute clockwise midgut volvulus with gangrene involving the distal ileum and cecum. The patient underwent hernia reduction, counterclockwise detorsion, resection of the gangrenous bowel, primary ileo-ascending colon anastomosis, and bowel repositioning according to the principles of the Ladd procedure. The patient's postoperative recovery was uneventful. This case highlights the importance of maintaining a high index of suspicion for congenital gastrointestinal anomalies in adults presenting with acute intestinal obstruction. It emphasizes that prompt diagnosis and timely surgical intervention are essential to prevent bowel loss and improve outcomes.

## Introduction

Internal hernias are a rare cause of intestinal obstruction, accounting for less than 1% of all cases. Among internal hernias, pericecal hernias constitute only a small proportion [1-3]. The simultaneous occurrence of congenital intestinal malrotation, pericecal internal hernia, and acute midgut volvulus is exceedingly rare, with very few cases reported in the literature, making preoperative diagnosis challenging for both clinicians and radiologists [3,4]. We present a case of acute small bowel obstruction with bowel gangrene in which emergency laparotomy revealed a pericecal internal hernia associated with congenital intestinal malrotation, with the duodenojejunal junction located on the right side. The patient was successfully managed with reduction of the incarcerated ileal loops, detorsion of the volvulus, resection of the gangrenous bowel, and primary anastomosis.

## Case presentation

**Figure 1:**
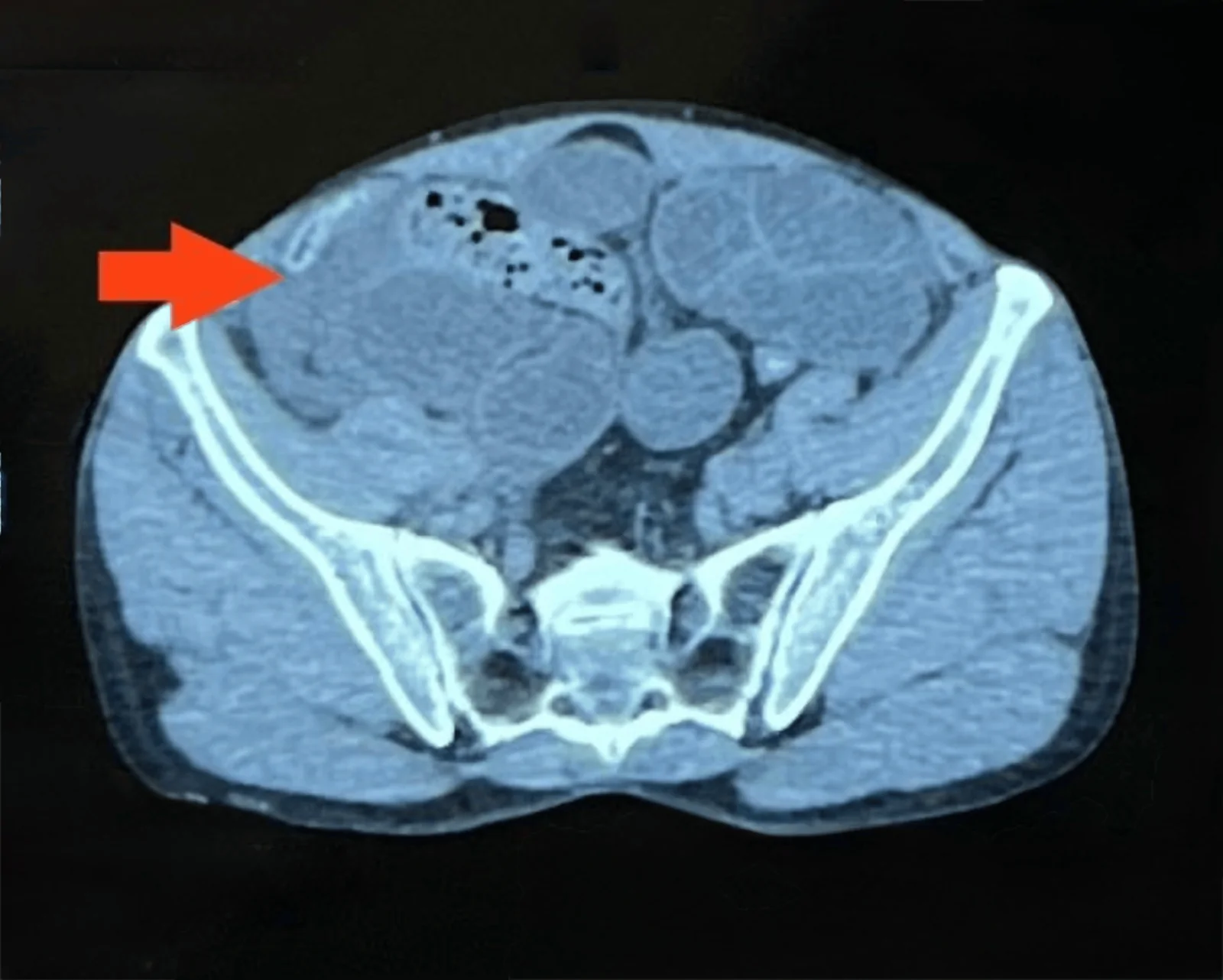
Computed tomography (CT) scan showing small bowel obstruction

A 57-year-old man presented to the emergency department with severe abdominal pain for three days, associated with progressive abdominal distension, constipation, obstipation, and vomiting. There was no other significant medical or surgical history. On examination, he was in hypovolemic shock. Initial management included nil per oral status, nasogastric tube decompression, and central venous pressure-guided fluid resuscitation, with maintenance of a mean arterial pressure >65 mmHg and urine output >1 mL/kg/hour. The abdominal exam showed diffuse tenderness with guarding and absent bowel sounds, while the rectal exam was unremarkable. Laboratory investigations showed leukocytosis 18 x 10^9^/L (normal range 4-11 x 10^9^/L), metabolic acidosis with a pH of 7.1 (normal range 7.35-7.45), and elevated serum lactate levels of 2.5 mmol/L (normal range 0.5-1.6 mmol/L). Contrast-enhanced computed tomography of the abdomen demonstrated dilated small bowel loops with a transition point near the terminal ileum, bowel wall thickening, and moderate intraperitoneal free fluid, suggestive of distal small bowel obstruction with bowel gangrene (Figure 1). Following adequate resuscitation, the patient underwent emergency exploratory laparotomy.

**Figure 2:**
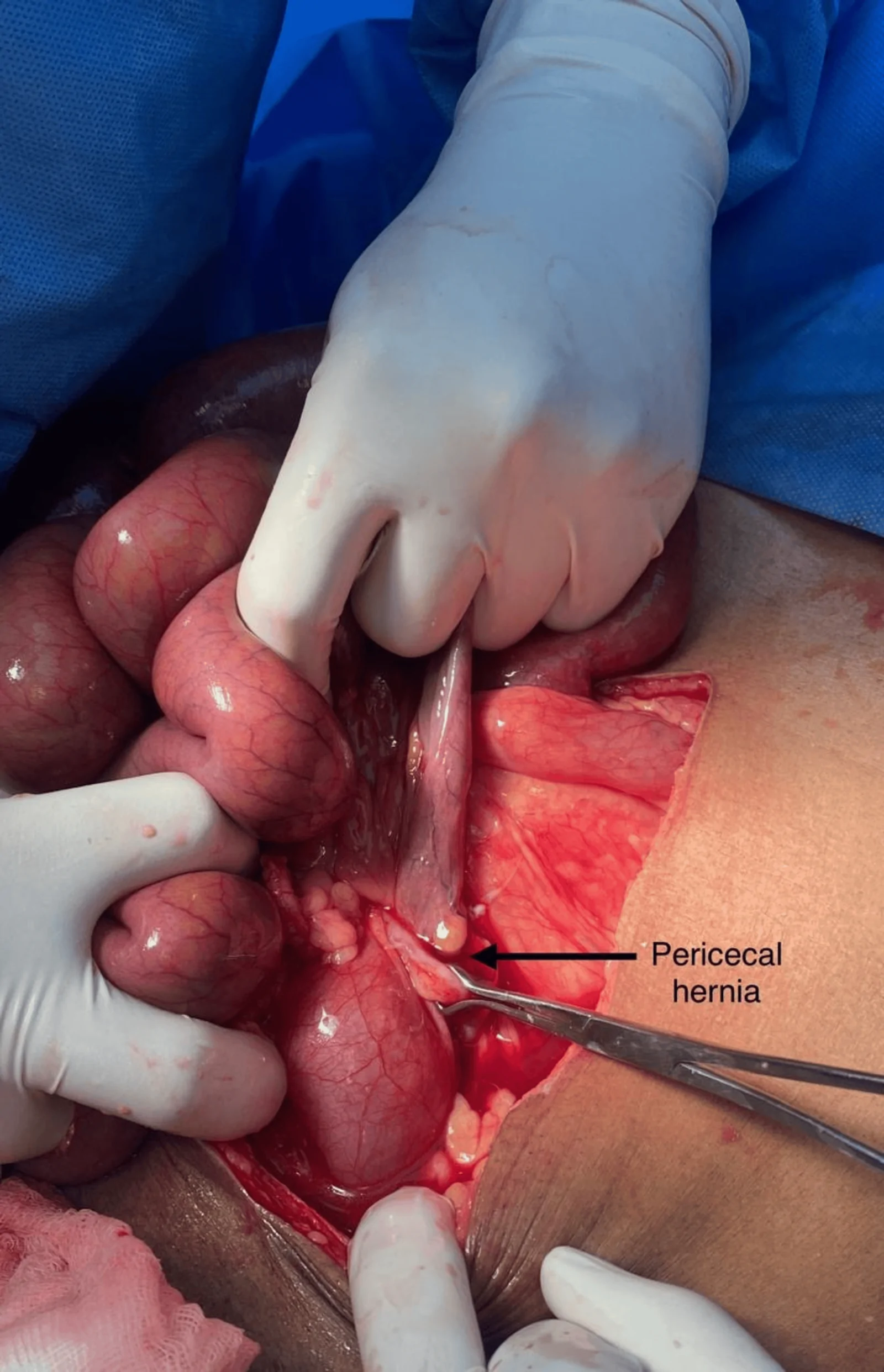
Intra operative picture showing pericecal hernia

Intraoperatively, incarcerated distal ileal loops and the cecum were found within a pericecal internal hernia (Figure 2).

The duodenojejunal flexure was identified on the right side, confirming congenital intestinal malrotation. The small intestine had undergone multiple clockwise rotations around the superior mesenteric artery, resulting in acute midgut volvulus. The herniated bowel was carefully reduced, followed by counterclockwise detorsion of the volvulus. After assessment of bowel viability, the gangrenous distal ileum and cecum were resected, and a primary ileo-ascending colon anastomosis was performed. The small bowel was positioned on the right side of the abdomen and the colon on the left in accordance with the principles of the Ladd procedure. The postoperative course was uneventful. Oral feeding was gradually resumed within 48 hours, and early mobilization was encouraged according to Enhanced Recovery After Surgery (ERAS) protocols. The patient was discharged on postoperative day seven.

## Discussion

Congenital intestinal malrotation is a rare developmental anomaly resulting from incomplete or abnormal 270° counterclockwise rotation and fixation of the primitive midgut around the superior mesenteric artery during embryogenesis. This leaves a narrow mesenteric base, predisposing the bowel to volvulus and vascular compromise. Although nearly 90% of cases present during infancy, adult presentation is uncommon, accounting for only 0.2-0.5% of the reported cases [1,3]. Adults may remain asymptomatic or present with nonspecific abdominal symptoms, often leading to delayed diagnosis until complications such as intestinal obstruction, midgut volvulus, ischemia, or perforation develop [3]. In this patient, congenital intestinal malrotation coexisted with a pericecal internal hernia and acute midgut volvulus, resulting in bowel gangrene. This exceptionally rare combination of two independent mechanisms of bowel obstruction likely accelerated vascular compromise, increasing diagnostic difficulty and emphasizing the need for a high index of suspicion in adults presenting with acute intestinal obstruction [3,4].

Internal hernias account for less than 1% of the general population but cause approximately 5-6% of small bowel obstruction cases, with pericecal hernias representing only 10-15% of internal hernias. These hernias arise from congenital peritoneal recesses adjacent to the cecum and may remain clinically silent until strangulation occurs, leading to bowel ischemia and gangrene if treatment is delayed [5,6]. Contrast-enhanced computed tomography is the imaging modality of choice for adult malrotation and may demonstrate an abnormally positioned duodenojejunal junction, reversed superior mesenteric artery-vein relationship, or the whirlpool sign of twisted mesenteric vessels. However, these classic features may be absent or overlooked in emergency settings. In our patient, CT demonstrated distal small bowel obstruction with bowel wall thickening and moderate free intraperitoneal fluid. These findings suggested intestinal strangulation, which warranted urgent surgical exploration, even though the congenital anomaly was not identified preoperatively [5,7].

Surgical management remains the definitive treatment for symptomatic intestinal malrotation regardless of age. The principles of the Ladd procedure include detorsion of the volvulus, division of obstructing congenital bands, widening of the mesenteric base, and placement of the small bowel on the right and colon on the left to reduce the risk of recurrent volvulus [8,9]. In this case, counterclockwise detorsion relieved the volvulus, reduction of the pericecal hernia restored bowel anatomy, and resection of the gangrenous distal ileum and proximal cecum with primary ileo-ascending colon anastomosis was required. Although laparoscopic surgery is increasingly used in stable patients without bowel ischemia, open laparotomy remains the preferred approach in the presence of bowel gangrene, severe bowel dilatation, or uncertain bowel viability [9,10].

Adult cases combining congenital intestinal malrotation, pericecal internal hernia, acute midgut volvulus, and bowel gangrene are exceptionally rare, particularly in patients beyond the fifth decade of life [4,6]. This case highlights that congenital gastrointestinal anomalies should remain an important differential diagnosis in older adults presenting with acute intestinal obstruction, even in the absence of previous abdominal surgery. The patient's uneventful postoperative recovery underscores the value of prompt diagnosis and timely surgical intervention. Although bowel resection was unavoidable because of established ischemia, early operative management prevented further life-threatening complications, and long-term outcomes are generally favourable when adequate bowel length is preserved and recurrent volvulus is prevented [11,12].

## Conclusions

Congenital intestinal malrotation associated with a pericecal internal hernia and acute midgut volvulus is an exceptionally rare cause of intestinal obstruction in adults. Early recognition, prompt surgical exploration, and definitive management are crucial to prevent bowel loss and life-threatening complications. This case highlights the importance of considering congenital gastrointestinal anomalies in adults presenting with acute intestinal obstruction, even in the absence of previous abdominal surgery.
